# An Evaluation of the Overall Utility of Measures of Functioning Suitable for School-Aged Children on the Autism Spectrum: A Scoping Review

**DOI:** 10.3390/ijerph192114114

**Published:** 2022-10-28

**Authors:** Maya Hayden-Evans, Benjamin Milbourn, Emily D’Arcy, Angela Chamberlain, Bahareh Afsharnejad, Kiah Evans, Andrew J. O. Whitehouse, Sven Bölte, Sonya Girdler

**Affiliations:** 1 Curtin Autism Research Group, School of Allied Health, Curtin University, Perth 6102, Australia; 2 Telethon Kids Institute, University of Western Australia, Perth 6009, Australia; 3 Autism CRC, Long Pocket, Brisbane 4850, Australia; 4 School of Allied Health, University of Western Australia, Perth 6009, Australia; 5 School of Psychological Science, University of Western Australia, Perth 6009, Australia; 6 Center of Neurodevelopmental Disorders (KIND), Centre for Psychiatry Research, Department of Women’s and Children’s Health, Karolinska Institutet & Stockholm Health Care Services, Region Stockholm, 104 31 Stockholm, Sweden; 7 Child and Adolescent Psychiatry, Stockholm Health Care Services, Region Stockholm, 104 31 Stockholm, Sweden

**Keywords:** adaptive behaviour, autism spectrum disorder, assessment, diagnosis, ICF Core Sets

## Abstract

A diagnosis of an autism spectrum condition (autism) provides limited information regarding an individual’s level of functioning, information key in determining support and funding needs. Using the framework introduced by Arksey and O’Malley, this scoping review aimed to identify measures of functioning suitable for school-aged children on the autism spectrum and evaluate their overall utility, including content validity against the International Classification of Functioning, Disability and Health (ICF) and the ICF Core Sets for Autism. The overall utility of the 13 included tools was determined using the Outcome Measures Rating Form (OMRF), with the Adaptive Behavior Assessment System (ABAS-3) receiving the highest overall utility rating. Content validity of the tools in relation to the ICF and ICF Core Sets for Autism varied, with few assessment tools including any items linking to Environmental Factors of the ICF. The ABAS-3 had the greatest total number of codes linking to the Comprehensive ICF Core Set for Autism while the Vineland Adaptive Behavior Scales (Vineland-3) had the greatest number of unique codes linking to both the Comprehensive ICF Core Set for Autism and the Brief ICF Core Set for Autism (6–16 years). Measuring functioning of school-aged children on the spectrum can be challenging, however, it is important to accurately capture their abilities to ensure equitable and individualised access to funding and supports.

## 1. Introduction

Autism spectrum conditions (hereinafter autism, aligning with the preferred language of the autistic community [1]) encompass a range of persistent neurodevelopmental outcomes, primarily characterised by altered social communication and social interaction behaviours, along with the presence of restricted or repetitive behaviours or interests [2,3]. According to these diagnostic criteria outlined in both the latest version of the Diagnostic and Statistical Manual of Mental Disorders [3] and the International Classification of Diseases [2], these traits must also have a considerable impact on an individual’s ability to function in educational, family, occupational, personal, social and/or other important domains and contexts [2,3]. Restrictions within these contexts can result in reduced social outcomes for children on the autism spectrum when compared to their peers [4]. Globally, the rate of autism diagnoses is increasing, with an estimated rate of at least 1 in 100 children diagnosed with autism [5,6], with the highest prevalence of autism seen among school-aged children [6,7].

During the school years, children spend a significant amount of time with their peers and are heavily influenced by their interactions with others. For young people on the spectrum, establishing and maintaining peer support networks may be hindered by their social and communication challenges [4], limiting their ability to develop the skills required to successfully navigate important developmental stages. In addition, school-aged children are driven to acquire complex competencies and develop independence across various areas of functioning, integrating their sense of self [8]. Given that impaired functioning is associated with an autism diagnosis, it is important to ensure that children on the spectrum are provided with sufficient supports to maximise their potential during their development.

In 2013, the Australian Government launched the National Disability Insurance Scheme (NDIS) following an inquiry into the previous disability support system, identifying the need for systemic change to improve outcomes for people with disabilities, including autism [9]. Delivered by the National Disability Insurance Agency (NDIA), the NDIS aspires to increase independence and promote social and economic engagement of individuals with significant and permanent disability by funding reasonable and necessary supports and services [10]. A substantial portion (65%) of children aged between seven and 14 who are currently accessing the NDIS are diagnosed with autism [11]. However, diagnosis alone provides limited information regarding an individual’s functioning and support needs which can vary significantly between individuals [12]. Therefore, the Australian guideline on autism assessment and diagnosis recommends that individuals on the spectrum receive a comprehensive needs assessment, including an assessment of functioning, to determine the level of support they require to participate effectively in their daily lives [13]. This approach aligns with other international guidelines that recommend assessment of functioning, including strengths, skills, impairments and needs, occurs across multiple contexts such as at home and at school [14,15,16].

Functioning, as it relates to health, is defined by the World Health Organization (WHO) [17] as “an umbrella term for body functions, body structures, activities and participation. It denotes the positive aspects of the interaction between an individual (with a health condition) and that individual’s contextual factors (environmental and personal factors)” (p. 8). The WHO’s framework for measuring health and disability, known as the International Classification of Functioning, Disability and Health (ICF), is a biopsychosocial model that can be used to organise information relating to functioning and disability [17]. Although useful for classifying information across the categories of Body Functions, Activities and Participation, and Environmental Factors, the comprehensiveness of the framework limits it’s utility in clinical settings [18]. However, recent publications suggest that the ICF can be used as a framework to guide the diagnostic and assessment process, in capturing the holistic nature of functioning and accounting for variability across contexts [13,19].

Despite recognising the importance of assessing functioning associated with autism, there is to date no universally accepted measure designed to assess the unique functional strengths and challenges of children on the spectrum [20]. At an individual level, understanding functioning is essential to planning and providing supports. The school years are a critical period of development, during which children are expected to comply with the demands and expectations of the classroom environment as well as in variable social contexts [21]. A benefit of the ICF is that it takes into account the unique environmental factors impacting a child’s functioning [17]. As highlighted by Bronfenbrenner’s bioecological theory, children develop and function across multiple contexts or ‘systems’, ranging from their immediate environments (microsystems) through to the broader contexts of society and culture (macrosystems) [21]. Understanding the functional impacts of autism across contexts is key in ensuring children have adequate opportunities to challenge themselves and develop their own identities. More broadly, understanding the functioning of individuals on the spectrum is important in developing and managing models of service delivery, allocating funding, and measuring support needs at a population level.

Previous research investigating the psychometric properties of measures has focused on younger children, up to the age of six [22], likely due to the emphasis on early assessment and intervention in autism. Other reviews of measures applicable for school-aged children on the spectrum focus on screening [23,24] and diagnostic measures [25]. However, given the shift towards assessing functioning alongside or within the diagnostic assessment process, there is a need to understand the utility of measures of functioning across age groups.

The Outcome Measures Rating Form (OMRF) [26] is a tool for evaluating the overall utility of outcome measures. The OMRF documents the focus of the measure, clinical utility, scale construction, standardisation, reliability, and validity. The overall utility of a measure is determined according to ease of availability, quality of psychometric properties, and level of clinical utility [26]. Clinical utility has been further conceptualised as consisting of four main components: (1) appropriate, including effectiveness and relevance; (2) accessible, including resource implications and procurement; (3) practicable, including functionality, suitability, and training or knowledge required; and (4) acceptable, from the perspective of clinicians, clients and society [27]. The COnsensus-based Standards for the selection of health Measurement INstruments (COSMIN) is an initiative aiming to improve the selection of health measurement instruments by facilitating evaluation of their content and measurement properties [28]. The COSMIN refers to three main quality domains: (1) reliability; (2) validity; and (3) responsiveness.

Content validity refers to how accurately the content of an instrument reflects the construct it intends to measure [29]. Validity is often measured indirectly using methods such as Rasch, factor analysis, or Item Response Theory; however, these methods alone may be limited in determining an instrument’s true validity [30]. Derived from the extensive ICF framework and developed using a rigorous, multi-phase research process with international data collection, the ICF Core Sets for Autism have established content validity and are well placed in providing a reference point in determining the content validity of existing measures evaluating the functioning in this population [31,32].

This review aimed to evaluate the overall utility of existing assessment of functioning measures suitable for assessing school-aged children on the spectrum. Research objectives included: identifying appropriate tools, investigating the components of overall utility using the OMRF [26] and determining their content validity against the ICF Core Sets for Autism [20]. This review provides a unique contribution to the current body of literature investigating measures of functioning in autism, summarising their overall utility, as well as providing new evidence highlighting their content validity for school-aged children on the spectrum. This review is expected to provide both an overview of the measures currently available to assess functioning in this age group, and guide clinicians when evaluating the suitability of existing measures for use with school-aged children, ranging between six and 16 years, on the spectrum.

## 2. Materials and Methods

### 2.1. Study Design

Scoping reviews can be used to explore a topic and synthesise the findings of existing research, identifying gaps in the current literature [33]. This review adopted the scoping review framework introduced by Arksey and O’Malley [33] and later refined by Levac et al. [34] and Daudt et al. [35]. The Preferred Reporting Items for Systematic reviews and Meta-Analyses extension for Scoping Reviews (PRISMA-ScR) is a checklist that was developed based on these existing frameworks to provide further clarity in reporting requirements of scoping reviews [36]. The scoping review framework and PRISMA-ScR were initially utilised to identify existing measures of functioning for school-aged children on the spectrum, then to evaluate their overall utility based on relevant research articles. This review undertook the following steps: (1) identifying the research question; (2) identifying relevant measures and studies; (3) selecting studies; (4) charting the data (including a methodological assessment of quality); and (5) collating, summarising, and reporting the results [33,34,35,36].

### 2.2. Identifying Relevant Tools and Studies

Scoping search strategies typically involve searching multiple sources, including both published and grey literature, to obtain a broad overview of relevant existing literature [34]. Since functioning is a broad concept that can be broken down into individual (e.g., body functions, body structures, activities) and contextual components (e.g., personal and environmental factors), the literature search was conducted in two phases: (1) search for relevant measures of functioning; and (2) search for studies evaluating the utility of those measures.

#### 2.2.1. Phase One

**Figure 1:**
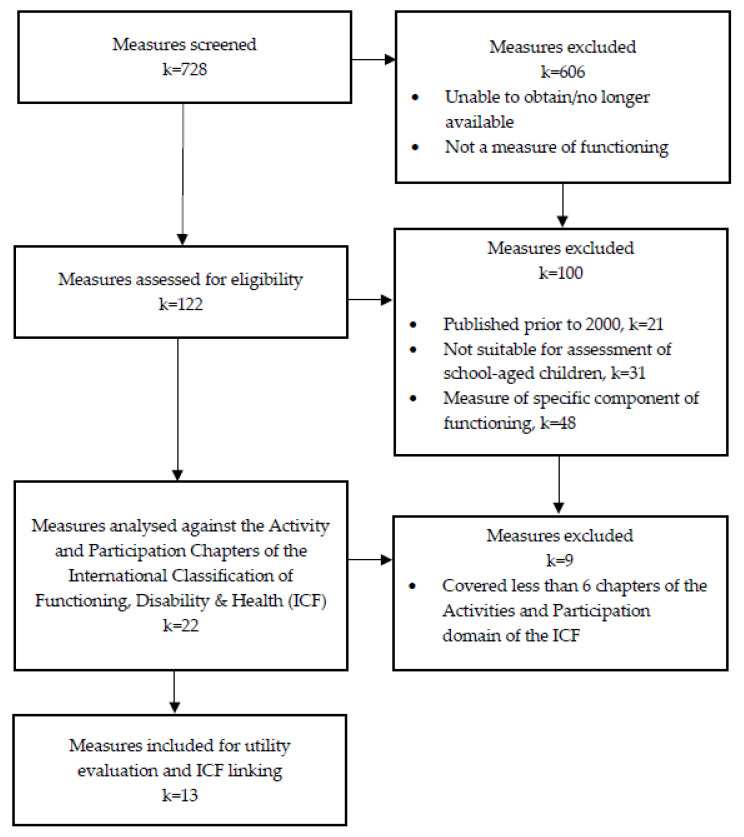
Phase One, study identification and screening process.

Measures were initially identified via internet searches, including Google, websites of major publishers (e.g., Pearson and Acer), reference books, catalogues of measures, and consultation with clinicians involved in assessing the functioning of individuals with neurodevelopmental conditions (Figure 1). Measures were eligible for inclusion in the review if they were: (1) available in English; (2) reflected at least six of the nine chapters included in the Activities and Participation domain of the ICF, to ensure inclusion of measures broadly assessing functioning; (3) were appropriate for use with individuals aged between six and 16 years, including measures that assessed either all or part of that range; and, (4) were published between January 2000 and June 2022. This timeframe was selected to ensure only the most recent versions of measures were included, aligning with current evidence-based practices. Measures were excluded if they: (1) only measured functioning in a specific population outside of neurodevelopmental conditions; (2) primarily measured impairment associated with a specific health condition (i.e., diagnostic tools); (3) had been superseded by a more recent version; or, 4) were no longer available online and/or in print. A shortlist of relevant measures was determined by two reviewers, guided by these criteria.

#### 2.2.2. Phase Two

**Figure 2:**
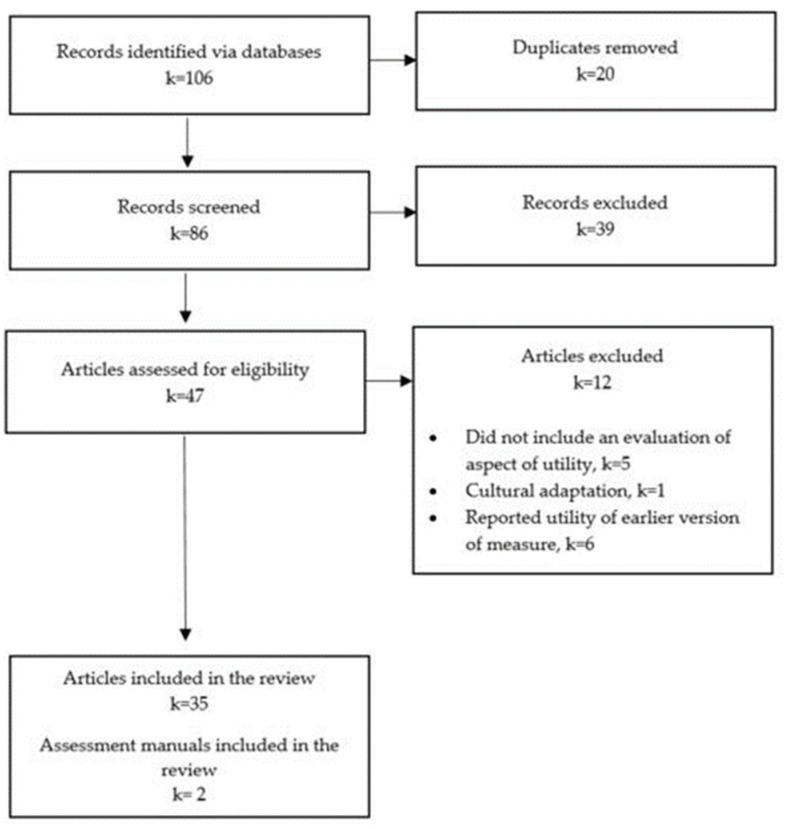
Phase Two, measures of functioning identification and screening process.

Targeted literature searches were conducted to identify articles reporting on aspects of overall utility, further defined in Table 1, of each measure identified in Phase One. The electronic databases ProQuest, Embase, CINAHL, and Scopus were used to search the literature for relevant articles published in English since 2000 (Figure 2). Search terms were grouped in relation to aspects of overall utility and the title of the measures, and searched for in the title or abstract of relevant resources. Combinations of search terms were truncated, exploded and adjusted with the assistance of a faculty librarian to meet the requirements of individual databases. Measure-specific search terms are included in Appendix A. Where applicable, the manuals of relevant measures were retrieved.

### 2.3. Selecting Studies

An iterative approach was utilised to ensure transparency and rigour of the scoping review process [34]. The inclusion and exclusion criteria were refined throughout the study selection process as familiarity with the research topic increased [33]. Studies were included if they reported on one or more aspects of overall utility of an included measure, and were peer-reviewed and available in full text. Studies were excluded if they only reported on the utility of an existing measure’s cultural adaptation.

### 2.4. Charting the Data

Data were extracted from the selected articles by two separate reviewers in line with the Arksey and O’Malley [33] framework. A data extraction table was developed and used to ensure a uniform data extraction process. Data from each of the articles were extracted in relation to the purpose of the study, study population, participant age, and methodological quality. Two reviewers independently evaluated the quality of the articles included in the review using the QualSyst checklists for assessing the quality of studies [37]. The QualSyst tool includes a 14-item checklist for evaluating quantitative studies and a 10-item checklist for evaluating qualitative studies. Each study was allocated a score represented as a percentage of 100 and a corresponding label indicating the study’s quality. As outlined by Kmet, Cook and Lee [37] in the user manual, a score of more than 80% indicates a strong study, 70–80% indicates good quality, 50–69% is adequate, and less than 50% indicates the study was of poor quality. Any inconsistencies in scores between the reviewers were resolved via discussion until total agreement was reached.

Two reviewers independently completed the OMRF [26] for each measure, taking into consideration the results of the targeted literature searches and the information included in the measures’ manuals, to evaluate their overall utility. Following this, any discrepancies between the two reviewers were discussed until consensus was reached. Using the OMRF [26], overall utility is assigned a descriptive rating, ranging from poor to excellent. An overall poor rating indicates poor clinical utility, the measure is not easily available, and has poor reliability and validity. An overall adequate rating indicates adequate to excellent clinical utility, the measure is easily available and has adequate to excellent reliability and validity. An overall excellent rating indicates adequate to excellent clinical utility, measure is easily available and has excellent reliability and validity.

In order to further determine the content validity of the included measures, specifically for autistic populations, meaningful concepts of each question or item included in the measure were independently linked by two reviewers to the ICF following the methodological rules outlined by Cieza et al. [38]. Using this methodology, items were first linked to the comprehensive ICF coding framework and later to specific ICF Core Sets for Autism, including the Comprehensive ICF Core Set and the Brief ICF Core Set for Autism (6–16 years) [20]. This process involved identifying the meaningful concepts in each item and linking these, as well as any examples, to the ICF. Meaningful concepts that were able to be linked to the ICF are referred to from this point forward as ‘codes’. Meaningful concepts that were determined ‘non-definable’ or ‘not covered’ in the ICF are not reported here. Consensus meetings were arranged to discuss any differences in the linking until total agreement was reached. Where the reviewers were unable to agree completely on a particular code for a meaningful concept, a third external reviewer experienced in ICF linking was consulted.

### 2.5. Collating, Summarising and Reporting the Results

PRISMA flow diagrams were developed and used to demonstrate the search process for Phases One and Two of this study. Key data extracted from the included articles were summarised and tabulated, including assessment tool characteristics, ICF Activities and Participation chapters covered, QualSyst ratings, and individual aspects of, as well as overall, utility. Descriptive statistics explaining the included measures’ coverage of both the comprehensive ICF Core Set for Autism and the Brief ICF Core Set for Autism (6–16 years) are also presented. A narrative synthesis of the available data was also conducted to summarise and highlight the key findings of the review.

## 3. Results

### 3.1. Identifying and Selecting Relevant Measures and Studies

Phase One identified 119 potential measures thorough searches of multiple sources. After applying the eligibility criteria listed above, 13 of these measures were deemed eligible for inclusion in the review. Targeted literature searches conducted in Phase Two returned a total of 106 abstracts. Once duplicates had been removed, 86 abstracts remained to be screened, and a total of 47 full-text articles were assessed for eligibility. The overall utility of the 13 eligible measures were assessed using a total of 35 original research articles, and two assessment manuals. The majority of articles investigating the utility of the measures were published by the authors of the measures themselves and no articles meeting the eligibility criteria were identified for either the Adaptive Behavior Assessment System (ABAS-3) or the Vineland Adaptive Behavior Scales (Vineland-3).

### 3.2. Measures of Functioning

An overview of the measures of functioning eligible for inclusion in this review, including a key for the abbreviations used in this section, is presented in Table 2.

Four of the measures (ABAS-3, AusTOMs-OT, LIFE-H and Vineland-3) have a broad age range and can be used to assess functioning of individuals across the lifespan. The COPM is suitable for anyone aged over eight years. The remainder of the measures were intended to assess child and youth populations under the age of 21 (CAPE/PAC, PEM-CY, PEDI-CAT and PEDI-CAT ASD, PEGS, and SCOPE), and one measure can be used for individuals aged between 4 and 6 years (CPQ). Measures were predominantly developed in Canada (COPM, CAPE/PAC, LIFE-H, PEM-CY, and PEGS) and the USA (ABAS-3, PEDI-CAT, PEDI-CAT ASD, SCOPE and Vineland-3).

Six of the measures were designed to be administered as self- or proxy-report questionnaires (ABAS-3, CPQ, LIFE-H, PEM-CY, PEGS, and ROPP). Similarly, the PEDI-CAT and PEDI-CAT ASD are administered as proxy-report computer adaptive tests. Three of the measures were intended to be completed by a health professional either as an interview (COPM) or by rating an individual’s functioning following interaction with them (AusTOMs-OT and SCOPE). The CAPE-PAC and Vineland-3 have a variety of administration options, meaning they can be completed as an interview or proxy-report questionnaire.

The number of ICF Activity and Participation chapters covered by each included measure ranged between six and nine, with an average of eight chapters being covered. All measures included at least one question relating to the chapters of *Domestic Life* and *Major Life Areas*. The ICF chapters with the lowest representation across the measures of functioning were *General Tasks and Demands* and *Communication*, with only nine of the 13 measures including a question linking to these chapters. The following measures included at least one question linking to each of the nine chapters of the Activities and Participation domain of the ICF: ABAS-3, LIFE-H, PEDI-CAT, PEDI-CAT ASD, and Vineland-3.

### 3.3. Methodological Quality

The methodological quality of the studies reporting on the psychometric properties of the measures included in the review are presented in Table 3. The quality of the studies, scored by two independent reviewers using the QualSyst checklists developed by Kmet, Cook and Lee [37] ranged between adequate (60%) and strong (100%).

### 3.4. Psychometric Properties

An overview of the psychometric properties available for each measure and an assessment of their overall utility is presented in Table 4. Overall utility ratings ranged from poor to excellent, with the ABAS-3 receiving the highest overall rating and the CAPE-PAC receiving the lowest overall rating on the OMRF. Information regarding at least one type of reliability and validity was available for all measures. Responsiveness was the least reported property, with this information only available for five of the 13 assessments.

General content validity of the measures ranged from adequate to excellent, however, none of the measures included in this review were developed with the specific intention of assessing functioning of individuals on the spectrum. The ‘Activities and Participation’ domain of the ICF was most commonly assessed by the measures, with all measures including codes linking to a chapter of this domain, ranging between 21% and 100% of total codes. Three assessments tools (CAPE-PAC, COPM and CPQ) solely assessed functioning classified as Activities and Participation. Coverage of the Body Function domain ranged between 0% and 79% of total codes, with the AusTOMs-OT having the greatest number of codes linking to chapters of this domain. Environmental Factors were assessed less frequently, ranging between 0% and 42% of total codes. Only four measures included codes linking to Environmental Factors (ABAS-3, PEM-CY, ROPP and SCOPE). More information regarding the distribution of codes across the domains and chapters of the ICF is included in Table 5.

Coverage of the comprehensive ICF Core Set for Autism ranged between 49% and 95%, with the ABAS-3 having the greatest total number of codes linking to this core set. Coverage of the Brief ICF Core Set for Autism (6–16 years) ranged between 35% and 73%, with the CAPE-PAC having the greatest total number of linked codes. However, all of these codes were linked to the Activities and Participation domain of the ICF. The percentages of total codes linking to the ICF Core Sets for Autism, both the Comprehensive and Brief (6–16 years), are presented in Table 6 for all included measures.

The percentages of unique codes linking to the comprehensive ICF Core Set for Autism and the Brief ICF Core Set (6–16 years) were also determined and are presented in Table 7. Overall, coverage of the Comprehensive ICF Core Set for Autism ranged between 11% and 61%, with the Vineland-3 having the greatest percentage of unique codes linking to this core set. Coverage of items relevant to the Brief ICF Core Set for Autism (6–16 years) was less, ranging between 5% and 58%, with the Vineland-3 again having the highest percentage of unique codes linking to this core set.

## 4. Discussion

This scoping review aimed to identify existing measures of functioning suitable for use with school-aged children on the spectrum and evaluate the quality of their psychometric properties, specifically content validity. The results of this review identify the limitations of current measures of functioning, highlighting the variability in content validity of these measures for school-aged children on the spectrum, and providing further evidence that, at present, a suite of measures is required to effectively assess functioning of school-aged children on the spectrum. Existing measures focus almost exclusively on functioning in relation to activity participation without exploring the impact of body functions or environmental factors on an individual’s ability to function. This review also emphasised other inconsistencies across existing measures of functioning, both in their overall utility, and their methods of administration.

In addition to the presence of key features such as repetitive and inflexible behaviour patterns and difficulties during social interactions, a diagnosis of autism requires that these features significantly impact an individual’s ability to function across a range of contexts, including at home, work and/or school [84]. Although impaired functioning is inherent to a diagnosis of autism, the methods of obtaining and interpreting this information remains unclear. The findings of this review indicate that there is no single measure that adequately covers all areas of functioning in which a school-aged child on the spectrum may experience difficulties, with environmental factors being particularly underrepresented in the assessment tools reviewed. These results are supported by the information outlined in current guidelines for assessing and diagnosing autism, highlighting the importance of obtaining information from multiple sources to build an accurate and comprehensive picture of how well a person is able to function in their everyday life, which includes multiple environmental contexts [13,16,85].

Developed following a rigorous process endorsed by the WHO, the ICF Core Sets provide an appropriate framework for organising information relating to functioning, and are also considered a suitable basis for development of tools to comprehensively measure functioning in particular populations [18]. The ICF Core Sets for other conditions, including hearing loss [86], spinal cord injuries [87] and cancer [88], have been operationalised through the development of outcome measures designed to assess functioning or intervention efficacy [86,87,88]. In their original form, the ICF Core Sets provide a standard for evaluating functioning in particular health conditions. However, the development of measures based on the ICF Core Sets can improve their clinical utility and promote the progression of more holistic, biopsychosocial approaches to measuring functioning.

Overall utility of measures of functioning included in this review varied significantly, ranging from poor to excellent according to the OMRF standards. The ABAS-3 received the highest overall OMRF rating, however, when evaluating the content validity of the ABAS-3 against the ICF Core Sets for Autism, it covered less than half of the items considered most relevant for individuals on the spectrum. Despite receiving a lower overall OMRF rating, the Vineland-3 covered a higher percentage of the items included in the ICF Core Sets for Autism. This suggests that although assessment of functioning tools may be considered psychometrically sound, their content validity may vary depending on the population they are being used to assess. This aligns with the findings of a similar evaluation of the content validity of measures suitable for use with younger children suspected of neurodevelopmental conditions [89]. It is important for clinicians to be aware of the suitability of these measures of functioning for specific populations, as this may influence their decision to select one tool over another. The COSMIN initiative aims to support this process by providing methodological guidelines to assist clinicians in selecting the appropriate assessment tool for their purpose [90]. In recent years, further work has been conducted to update earlier COSMIN guidelines, providing greater clarity around the selection of tools based on content validity [91]. Poor content validity can influence other psychometric properties, reducing the quality of overall reliability, validity and responsiveness, suggesting that establishing content validity should be prioritised before other psychometric properties [91]. A factor to consider in the interpretation of the results presented in this review is that full assessment item banks were coded to the ICF. Some assessments, such as the Vineland-3, use basal and ceiling thresholds to determine which items are presented for scoring [50], meaning that not all items included in the full item bank are presented during an assessment, potentially reducing the content validity of an assessment in clinical application. Similarly, the PEDI-CAT and PEDI-CAT ASD are administered via Computer Adaptive Test, presenting users with questions based on their previous responses and therefore not including all items evaluated in this review [67].

The method of administration varied among the measures of functioning included in this review, with the majority being clinician-administered or proxy-report. There are very limited options available for children to self-report, providing their own perspectives and priorities for functioning. Since autism is a complex condition influenced by a variety of internal and external factors, using a variety of assessments to obtain information from multiple perspectives can again help to provide a more holistic understanding of an individual’s functional challenges and abilities [13]. In isolation, a clinician’s perspective may not adequately reflect the functional impact of autism in a home or school environment, and proxy-reporting caregivers may not have adequate knowledge or understanding to effectively report impacts on functioning that may be better observed by a clinician in a standardised environment [92,93]. It is important to also consider the context in which functioning is being assessed and the supports that may or may not be in place during the assessment [93]. There is inconsistency in the current measures regarding the ways in which functioning is assessed; some tools consider the person’s abilities with supports in place (e.g., PEDI-CAT/PEDI-CAT ASD) while others do not (e.g., ABAS-3 and Vineland-3). Inconsistencies such as these can lead to confusion regarding an individual’s true functioning and support needs, which may be better assessed by measures specifically developed to explore these needs [94]. Across measures included in this review there is limited consideration of the impact of cultural factors on functioning, with the majority of these assessments being developed and tested in Canada or the USA. In addition, there is a paucity of research investigating the utility of these tools outside of the teams who developed them. A previous review of adaptive behaviour scales by Floyd and colleagues [93] evaluated the psychometric evidence for a variety of scales, including earlier versions of the Vineland and ABAS, however, only considered the evidence available in the manuals of these tools. Similarly, during this review, no recently published peer-reviewed articles reporting on aspects of overall utility of either the Vineland-3 or ABAS-3 were identified, only the information provided by the publishers in the user manual.

Historically, the biomedical model of health and disability has concentrated on impairment, attributing disability to a particular health condition, with interventions focussed on preventing or treating the condition with the goal of ‘normalising’ functioning [95]. In contrast, the social model of disability views disability as a consequence of social, environmental and attitudinal barriers, secondary to the condition itself [96]. More recently, autism and other neurodevelopmental conditions have been conceptualised under the neurodiversity paradigm, which aligns in some regards with the social model of disability, considering disability to be the consequence of external rather than internal factors [97]. The neurodiversity paradigm re-frames the differences seen in neurodiverse individuals as strengths that may be used to support interventions and positively influence functioning [19]. As views of neurodiversity continue to evolve, so too does the need for measurement tools to accurately reflect the current contexts in which individuals on the spectrum live and function [19]. Researchers are beginning to acknowledge the importance of involving consumers in the research process, increasingly using methods of co-production to incorporate the views of the target population [98].

The authors acknowledge that there are limitations to this review. The inclusion criteria specified that only studies published in English were eligible for inclusion, which may account for the lack of cultural diversity represented among the measures and studies. In addition, only articles investigating elements of overall utility of the most recent version of the measure were considered which may influence the availability of psychometric information for measures where this has been established in earlier versions. Finally, although a comprehensive approach was taken to ensure a broad search of the literature, it is possible that these methods may not have captured every available article reporting on the overall utility of the included measures.

## 5. Conclusions

This review contributes to the existing literature by providing a useful summary of the psychometric properties of measures of functioning that can be used by researchers and clinicians to facilitate the selection of suitable measures for assessing functioning of school-aged children on the autism spectrum. Effectively assessing functioning of school-aged children on the spectrum is increasingly important in both the Australian and international contexts given the shift towards disability support systems allocating funding based on level of functioning and support needs. For individuals on the spectrum, functioning can vary significantly, highlighting the need for reliable and valid methods of assessment that are capable of identifying the unique strengths and challenges of this population. There are a number of factors which should be considered when selecting a measure of functioning, including the purpose of the assessment, the population it is assessing, and the complete range of psychometric properties, including content validity. This review not only synthesises the properties of existing measures, but adds a comprehensive evaluation of the content validity of these measures for use with school-aged children on the spectrum. Further research in this area is required to ensure measures of functioning align with contemporary views of disability and are developed in collaboration with those most likely to benefit from them. Future research may seek to develop and evaluate holistic assessment of functioning tools based on the ICF Core Sets for Autism, with input from individuals on the spectrum and their families.

## Figures and Tables

**Table 1:** The COnsensus-based Standards for the selection of health Measurement INstruments (COSMIN) definitions of domains and psychometric properties [28] (Note. Adapted from: “COSMIN methodology for systematic reviews of Patient-Reported Outcome Measures (PROMs)” by Mokkink, L.B.; Terwee, C.B.; Patrick, D.L.; Alonso, J.; Stratford, P.W.; Knol, D.L.; Bouter, L.M.; de Vet, H.C., 2012, Amsterdam Public Health Research Institute: BT Amsterdam, p. 11–12 (https://cosmin.nl/wp-content/uploads/COSMIN-syst-review-for-PROMs-manual_version-1_feb-2018.pdf)).

Term	Definition
**Reliability**	**The Consistency with Which a Measure Produces the Same Results.**
Internal consistency	The level of correlation between items.
Reliability - Test–retest - Inter-rater - Intra-rater	The level of discrepancy in measurements resulting from actual differences between individuals.
Measurement error - Test–retest - Inter-rater - Intra-rater	Systematic and random errors that are not a consequence of actual changes in the construct being assessed.
**Validity**	**The level at which a measure actually evaluates the construct(s) it is intended to measure.**
Content validity - Face validity	How accurately the content of a measure reflects the construct being evaluated.
Construct validity - Structural validity - Hypotheses testing - Cross-cultural validity	The level of accuracy with which the measure evaluates what it is intended to.
Criterion validity - Concurrent validity - Predictive validity	The accuracy with which the scores of a measure adequately reflect the ‘gold standard’.
**Responsiveness**	**How accurately a measure is able to detect change over time in the construct being evaluated.**
Responsiveness	The relationship between unobservable traits and how they present.

**Table 2:** Characteristics of included measures of functioning.

Measure	Abbreviation	Year	Authors	Format	Number of Items	Age Range (Years)
Adaptive Behavior Assessment System (3rd Edition) USA	ABAS-3	2015	Harrison & Oakland [39]	Self- or proxy-report questionnaire	Parent-form (0–5 years) = 241	0–89
					Parent-form (5–21 years) = 232	
Assessment of Life Habits Canada	LIFE-H	2014	Noreau, Fougeyrollas & Vincent	Self- or proxy-report questionnaire	Long-form = 242	Any
					Short-form = 77	
Australian Therapy Outcome Measures for Occupational Therapy (3rd Edition) Australia	AusTOMs-OT	2004	Unsworth & Dunscombe [40]	Occupational therapist-rated scales	12	Any
Canadian Occupational Performance Measure Canada	COPM	2000	Law, Baptiste, Carswell, McColl, Polatjko & Pollock [41]	Interview by occupational therapist	9	8+
Children’s Assessment of Participation and Enjoyment and Preferences for Activities of Children	CAPE/PAC	2005	G. King, Law, S. King, Hurley, Rosenbaum, Hanna, Kertoy & Young [42]	Interview by health professional or self-report	Total = 110	6–21
					CAPE = 55	
					PAC = 55	
Children’s Participation Questionnaire Israel	CPQ	2010	Rosenberg, Jarus & Bart [43]	Proxy-report questionnaire	44	4–6
Participation and Environment Measure for Children and Youth Canada	PEM-CY	2010	Coster, Law & Bedell [44]	Proxy-report questionnaire	45	5–17
Pediatric Evaluation of Disability Inventory—Computer Adaptive Test USA	PEDI-CAT	2012	Haley, Coster, Dumas, Fragala-Pinkham & Moed [45]	Proxy-report computer adaptive test	Item bank = 276	0–21
					Content-balanced version ≤ 30 items)	
					Speedy version ≤ 15 items	
Pediatric Evaluation of Disability Inventory—Computer Adaptive Test (Autism Spectrum Disorder) USA	PEDI-CAT ASD	2019	Haley, Coster, Dumas, Fragala-Pinkham, Moed, Kramer, Ni, Feng, Kao & Ludlow [46]	Proxy-report computer adaptive test	301	0–21
Perceived Efficacy and Goal Setting System Canada	PEGS	2004	Missiuna, Pollock & Law [47]	Child self-report and parallel proxy-report questionnaires	24	5–9
Rating of Perceived Participation Sweden	ROPP	2007	Sandström & Lundin-Olsson [48]	Self-report questionnaire	22	Unspecified
Short Child Occupational Profile USA	SCOPE	2008	Bowyer, Kramer, Ploszaj, Ross, Schwartz, Kielhofner & Kramer [49]	Occupational therapist-rated performance measure	25	0–21
Vineland Adaptive Behavior Scales (3rd Edition) USA	Vineland-3	2016	Sparrow, Cicchetti & Saulnier [50]	Interview by health professional; proxy-report form (available for parent/caregiver or teacher)	Comprehensive interview/parent-form = 502	0–90
					Comprehensive teacher-form = 333	
					Domain interview = 195	
					Domain parent-form = 180	
					Domain teacher-form = 149	

**Table 3:** Descriptions and QualSyst ratings of studies evaluating the psychometric properties of included measures of functioning.

Measure ^1^	Reference	Study Purpose	Study Population	Age ^2^ (Years)	QualSyst Score
ABAS-3	Harrison & Oakland, 2015 [39]	To describe the psychometric properties of the ABAS-3 based on standardised and clinical samples.	*n* = 265 children and adults from standardisation sample	R: 0–84 Children R: 0–18 M: 4.6 (SD 4.7)	N/A
			Clinical group: Autism Sample 1: *n* = 51 pre-school aged children	Autism Sample 1 R: 24–71 months M: 54 months (SD 11.2)	
			Sample 2: *n* = 37 school-aged children	Autism Sample 2 R: 5–20 M: 10.6 (SD 4.0)	
AusTOMs	Scott, Unsworth, Fricke & Taylor, 2006 [51]	To determine retest reliability, interrater and intrarater reliability of the AusTOMS OT—Self Care scale	*n* = 7 occupational therapists	R: 22–44 M: 32	19/22 86% Strong
	Unsworth, 2005 [52]	To determine the sensitivity of the AusTOMS-OT scales in detecting change to client status over time.	*n* = 466 (*n* = 106 children)	Children R: 0–18 M: 10.4	18/22 82% Strong
	Unsworth, Coulson, Swinton, Cole & Sarigiannis, 2014 [53]	To establish the minimal clinically important difference for four domains of the AusTOMs-OT.	*n* = 787 clients of a home-based therapy service	R: 18–101 M: 71.5 (SD 14.7)	21/24 88% Strong
	Unsworth, Timmer & Wales, 2018 [54]	To investigate inter-rater and intra-rater reliability of occupational therapists using the AusTOMs-OT, and level of agreement for all AusTOMs-OT scales, including test–retest reliability, measurement error, and the error range.	*n* = 31 occupational therapists	M: 38.0 (SD 10.0)	23/24 96% Strong
	Unsworth, Duckett, Duncombe, Perry, Skeat & Taylor, 2004 [55]	To investigate the construct (convergent) validity of the AusTOMs in comparison to EuroQuol-5D	*n* = 205 occupational therapy (67), physiotherapy (110), and speech pathology (28) clients	Unspecified	19/22 86% Strong
CAPE/PAC	Brown & Thyer, 2019 [56]	To examine the convergent validity between the Children’s Leisure Assessment Scale and CAPE-PAC.	*n* = 40 healthy Australian children	M: 9.2 (SD 2.0)	21/22 95% Strong
	G.A King, Law, S. King, Hurley, Hanna, Kertoy & Rosenbaum, 2007 [57]	To determine construct validity of the CAPE and PAC.	*n* = 427 children with physical functional limitations	R: 6–15	20/22 91% Strong
	Potvin, Snider, Prelock, Kehayia & Wood-Dauphinee, 2013 [58]	To determine the psychometric properties of the CAPE/PAC for children with high functioning autism.	*n* = 61 (*n* = 30 children with high functioning autism; *n* = 31 typically developing peers)	Autism sample R: 7–13	19/22 86% Strong
COPM	Eyssen, Beelen, Dedding, Cardol & Dekker, 2005 [59]	To assess the reliability and inter-rater agreement of the COPM.	*n* = 95 occupational therapy clients	R: 19–80 M: 47.0 (SD 15.0)	24/26 92% Strong
	McColl, Paterson, Davies, Doubt & Law, 2000 [60]	To determine the validity and community utility of the COPM.	*n* = 61 individuals with a disability	R: 18->75	22/22 100% Strong
	Tuntland, Aaslund, Langeland, Espehaug & Kjeken, 2016 [61]	To determine the validity, responsiveness, interpretability and feasibility of the COPM for home-dwelling older adults.	*n* = 225 older adults	M: 80.8	21/22 95% Strong
	Verkerk, Wold, Louwers, Meester-Delver & Nollet, 2006 [62]	To determine the inter-rater agreements, construct and criterion validity of the COPM in parents of children with disabilities.	*n* = parents of 80 children	Parents’ age: R: 24–48 M: 35.0 (SD 5.0) Children’s age R: 1–7.5 M: 3.70 (SD 1.80)	22/24 91% Strong
	Cusick, Lannin & Lowe, 2007 [63]	To determine internal consistency, content and construct validity, responsiveness and impact of half scores of the adapted COPM (for children).	*n* = 42 children with spastic hemiplegic cerebral palsy	R: 2–7 M: 3.90	23/26 88% Strong
CPQ	Rosenberg, Jarus & Bart, 2010 [43]	To develop and test the psychometric properties of the CPQ.	*n* = 480 (*n* = 231 children with developmental difficulties; *n* = 249 typically developing children)	Developmental difficulties sample: M: 5.2 (SD 0.7) Typically developing sample: M: 5.1 (SD 0.7)	22/22 100% Strong
LIFE-H	Noreau, Desrosiers, Robichaud, Fougeyrollas, Rochette & Viscogliosi, 2004 [64]	To document the reliability of the LIFE-H.	*n* = 84 individuals with physical disabilities	M: 78.0 (SD 8.2)	20/22 91% Strong
	Noreau, Lepage, Boissiere, Picard, Fougeyrollas, Mathieu, Desmarais & Nadeau, 2007 [65]	To examine the psychometric properties of the LIFE-H and draw a profile of the level of participation of children aged 5–13 years with impairments.	*n* = 94 parents of children with disabilities *n* = 29 experts (content validity panel)	Children’s age: M: 8 years 10 months (SD 2 years 6 months)	20/22 91% Strong
PEDICAT	Dumas & Fragala-Pinkham, 2012 [66]	To examine concurrent validity of the PEDI-CAT Mobility domain with the PEDI Functional Skills (FS) Mobility Scale, evaluate item-specific reliability between the PEDI-CAT Mobility domain and PEDI FS Mobility Scale, and assess score distributions for floor and ceiling effects.	*n* = 35 parents	Children’s age: R: 3.93–19.87 M: 11.49 (SD 4.89)	15/20 75% Good
	Dumas, Fragala-Pinkham, Haley, Ni, Coster, Kramer, Kao, Moed & Ludlow, 2012 [67]	To assess discriminant validity of the PEDI-CAT and assess test–retest reliability, administration time, and obtain parental feedback about the tool.	*n* = 102 (*n* = 50 parents of children with disabilities; *n* = 52 parents of children without disabilities; *n* = 25 retest sample)	Children’s age: R: 3–20 M: 10.30 (SD 4.64)	20/22 91% Strong
	Dumas, Fragala-Pinkham, Rosen & O’Brien, 2017 [68]	To assess construct (convergent and divergent) validity of the PEDI-CAT in children with complex medical conditions.	*n* = 110 children	R: 0.22–21.93 M: 5 (SD 5.65)	17/20 85% Strong
	Hayley, Coster, Dumas, Fragala-Pinkham, Kramer, Ni, Tian, Kao, Moed & Ludlow, 2011 [69]	Assess the accuracy and precision of PEDI-CAT item banks for ages 0–21 years.	*n* = 2822 (*n* = 617 young people with a disability; *n* = 2205 typically developing young people)	Disability sample: M: 11 years 8 months (SD 4.7) Typically developing sample: M:10 years 1 month (SD 6.1)	19/20 95% Strong
	Shore, Allar, Miller, Matheney, Snyder & Fragala-Pinkham, 2019 [70]	To investigate the construct validity and test–retest reliability of the PEDI-CAT for children with cerebral palsy (CP).	*n* = 101 children with CP	R: 6–20 M: 11.9 (SD 3.70)	22/22 100% Strong
	Shore, Allar, Miller, Matheney, Snyder & Fragala-Pinkham, 2017 [71]	To determine the discriminant validity of the PEDI-CAT according to the Gross Motor Function Classification System and Manual Ability Classification System in children with CP.	*n* = 101	R: 6–20 M: 11.9 (SD 3.70)	22/22 100% Strong
PEDI-CAT ASD	Coster, Kramer, Tian, Dooley, Liljenquist, Kao & Ni, 2016 [72]	To evaluate the structural validity of the PEDI-CAT for children and youth with symptoms of ASD.	*n* = 365	R: 3–21 M: 11.9 (SD 4.67)	19/20 95% Strong
	Kramer, Coster, Kao, Snow & Orsmond, 2012 [73]	To evaluate the applicability, representativeness, and comprehensiveness of the PEDI-CAT for children and youth with ASD.	*n* = 20 professionals *n* = 18 parents representing *n* = 21 children and youth with ASD	Children’s age: R: 3 years 8 months—17 years 11 months M: 9 years 8.5 months (SD 45.3 months)	16/20 80% Good
	Kramer, Liljenquist & Coster, 2016 [74]	To explore test–retest reliability of the PEDI-CAT for ASD and concurrent validity with Vineland-II.	*n* = 39 parents	Children’s age: R: 10 years 3 months–18 years 10 months M: 14 years 10 months (SD 2 years 8 months)	16/20 80% Good
PEGS	Missiuna & Pollock, 2000 [75]	To pilot a measure and process providing young children with the opportunity to assess their performance on daily tasks and aid goal setting.	*n* = 37 children and parents	Children’s age: R: 5–9	19/22 86% Strong
	Missiuna, Pollock, Law, Walter & Cavey, 2006 [76]	To determine whether children with a disability could self-report their competence performing everyday activities, and establish whether these self-reports could be used to establish and prioritise occupational therapy intervention goals.	*n* = 117	R: 6–10 M: 7.7	21/22 95% Strong
PEM-CY	Coster, Bedell, Law, Khetani, Teplicky, Liljenquist, Gleason & Kao, 2011 [77]	To examine the psychometric properties of the PEM-CY.	*n* = 576 caregivers of children and young people	Children’s age: R: 5–17 M: 11 (SD 3.1)	20/22 91% Strong
	Coster, Law, Bedell, Khetani, Cousins & Teplicky, 2011 [78]	To describe the conceptual foundation of the PEM-CY	N/A	N/A	12/20 60% Adequate
	Khetani, Marley, Baker, Albrecht, Bedell, Coster, Anaby & Law, 2014 [79]	To examine the concurrent validity and utility of the PEM-CY for Health Impact Assessment in non-urban sustainable development projects affecting children with disabilities.	*n* = 89 parents of children and youth with disabilities	M: 11.91 (SD 3.36)	22/22 100% Strong
ROPP	Sandstrom & Lundin-Olsson, 2007 [48]	To develop a questionnaire for self-rated perceived participation and evaluate its psychometric properties.	*n* = 85	R: 23–79 M: 55.5 (SD 13.3)	21/22 95% Strong
	Noonan, Kopec, Noreau, Singer, Chan, Masse & Dvorak, 2009 [80]	To determine the content validity of measures of participation by linking to the ICF.	N/A	N/A	15/16 94% Strong
SCOPE	Bowyer, Kramer, Kielhofner, Maziero-Barbosa & Girolami, 2007 [81]	To examine the reliability and validity of the SCOPE.	*n* = 36	R: 2–21 M: 3	21/22 95% Strong
	Kramer, Bowyer, Kielhofner, O’Brien & Maziero-Barbosa, 2009 [82]	To assess how practitioners performed using a revised version of the SCOPE and the effect of revisions on practitioner rating behaviours.	*n* = 39 practitioners reporting on *n* = 168 paediatric clients.	Children’s age: R: 6 months–15 years 8 months M: 4 years 10.96 months (SD 35.27 months)	21/22 95% Strong
	Bowyer, Lee, Kramer, Taylor & Kielhofner, 2012 [83]	To determine the clinical utility of the SCOPE.	*n* = 21 practitioners	Not reported	17/20 85% Strong
Vineland-3	Sparrow, Cicchetti & Saulnier, 2016 [50]	User manual, describing psychometric properties of the Vineland-3.	*n* = 2560 (normative sample)	R:0–90	N/A

^1^ ABAS-3, Adaptive Behavior Assessment System, 3rd edition; AusTOMs-OT, Australian Therapy Outcome Measures for Occupational Therapy, 3rd edition; CAPE-PAC, Children’s Assessment of Paticipation and Enjoyment and Preferences for Activities of Children; COPM, Canadian Occupational Performance Measure; CPQ, Children’s Participation Questionnaire; LIFE-H, Assessment of Life Habits; PEDI-CAT, Pediatric Evaluation of Disability Inventory—Computer Adaptive Test; PEDI-CAT (MD), PEDI-CAT with mobility device; PEDI-CAT ASD, PEDI-CAT module for autism spectrum disorder; PEGS, Perceived Efficacy Goal Setting System; ROPP, Rating of Perceived Participation; SCOPE, Short Child Occupational Profile; Vineland-3, Vineland Adaptive Behaviour Scales, 3rd edition; ^2^ R, range; M, mean; SD, standard deviation.

**Table 4:** Characteristics of included assessment of functioning tools.

Measure ^1^	Reliability		Validity			Responsiveness	Overall Utility
	Internal Consistency	Reliability	Content Validity	Construct Validity	Criterion Validity		
ABAS-3	Excellent 0.96–0.99	Test-retest Excellent 0.82	Excellent	Excellent	Excellent	-	Excellent
		Inter-rater Adequate to excellent 0.67–0.85					
		Alternate-forms Adequate to excellent 0.79–0.95					
AusTOMs-OT	-	Test–retest Adequate to excellent 0.616–0.960	Excellent	Adequate	Adequate	Excellent	Adequate
		Inter-rater Adequate >0.70					
		Intra-rater Adequate >0.74					
COPM	Excellent 0.86–0.88	Test–retest Adequate 0.67–0.69	Excellent	Excellent	Adequate	Adequate	Adequate
		Inter-rater Excellent 0.80					
CAPE/PAC	CAPE Poor to adequate 0.42–0.77	Test–retest Poor to excellent 0.55–0.81	Adequate	Adequate	Adequate	Adequate	Poor
	PAC Adequate to excellent 0.76–0.84	Test–retest (ASD) Poor to adequate 0.196–0.758					
CPQ	Adequate to excellent 0.79–0.90	Test–retest Excellent 0.84–0.90	Adequate	Adequate	Adequate	Adequate	Adequate
LIFE-H	-	Test–retest Excellent 0.95	Excellent	Adequate	-	-	Adequate
		Inter-rater Adequate to excellent 0.78–0.89					
		Intra-rater Adequate >0.75					
PEDI-CAT	Poor to excellent 0.3390–1.00	Test–retest Excellent 0.90–0.99	Excellent	Adequate	Adequate	Adequate	Adequate
		Inter-rater Excellent 0.83–0.89					
PEDI-CAT ASD	-	Test–retest Excellent 0.86–0.92	Excellent	Adequate	Adequate	-	Adequate
PEGS	Adequate 0.795	Inter-rater Poor 0.261–0.307	Excellent	Adequate	Adequate	-	Adequate
PEM-CY	Adequate to excellent 0.67–0.80	Test–retest Poor to excellent 0.58–1.00	Excellent	Adequate	Adequate	-	Adequate
ROPP	Excellent 0.90	Test–retest Excellent 0.97	Adequate	Adequate	Adequate	-	Adequate
SCOPE	Excellent 0.90	Inter-rater Adequate to excellent 0.64–0.83	Excellent	Adequate	-	-	Adequate
Vineland-3	Excellent 0.90–0.98	Test–retest Adequate to excellent 0.73–0.92	Excellent	Adequate	Adequate	-	Adequate
		Inter-rater Adequate to excellent 0.70–0.81					

^1^ ABAS-3, Adaptive Behavior Assessment System, 3rd edition; AusTOMs-OT, Australian Therapy Outcome Measures for Occupational Therapy, 3rd edition; CAPE-PAC, Children’s Assessment of Paticipation and Enjoyment and Preferences for Activities of Children; COPM, Canadian Occupational Performance Measure; CPQ, Children’s Participation Questionnaire; LIFE-H, Assessment of Life Habits; PEDI-CAT, Pediatric Evaluation of Disability Inventory—Computer Adaptive Test; PEDI-CAT (MD), PEDI-CAT with mobility device; PEDI-CAT ASD, PEDI-CAT module for autism spectrum disorder; PEGS, Perceived Efficacy Goal Setting System; ROPP, Rating of Perceived Participation; SCOPE, Short Child Occupational Profile; Vineland-3, Vineland Adaptive Behaviour Scales, 3rd edition.

**Table 5:** Distribution of codes linked to the International Classification of Functioning, Disability and Health [17] components and chapters.

	Measures of Functioning ^1^														
	ABAS-3	AusTOMs-OT	CAPE-PAC	COPM	CPQ	LIFE-H	PEDI-CAT	PEDI-CAT (MD)	PEDI-CAT ASD	PEDI-CAT ASD (MD)	PEGS	PEM-CY	ROPP	SCOPE	Vineland-3
Total codes	256	99	59	36	47	98	270	213	315	258	26	112	29	57	648
**Body functions** **(%)**	**22** **(8)**	**78** **(79)**	**0**	**0**	**0**	**1** **(1)**	**17** **(6)**	**17** **(8)**	**19** **(7)**	**19** **(7)**	**2** **(8)**	**3** **(3)**	**0**	**6** **(11)**	**117** **(18)**
Mental	21 (95)	10 (13)	0	0	0	1 (100)	16 (94)	16 (94)	18 (95)	18 (95)	1 (50)	0	0	4 (67)	107 (91)
Sensory and pain	0	12 (15)	0	0	0	0	0	0	0	0	0	0	0	0	4 (3)
Voice and speech	1 (5)	9 (12)	0	0	0	0	0	0	0	0	0	0	0	0	1 (1)
Cardiovascular, haematological, immunological and respiratory	0	9 (12)	0	0	0	0	0	0	0	0	0	3 (100)	0	0	0
Digestive, metabolic and endocrine	0	9 (12)	0	0	0	0	1 (6)	1 (6)	1 (5)	1 (5)	0	0	0	0	1 (1)
Genitourinary and reproductive	0	9 (12)	0	0	0	0	0	0	0	0	0	0	0	0	1 (1)
Neuromusculoskeletal and movement-related	0	11 (14)	0	0	0	0	0	0	0	0	1 (50)	0	0	2 (33)	3 (3)
Skin and related structures	0	9 (12)	0	0	0	0	0	0	0	0	0	0	0	0	0
**Activities and participation** **(%)**	**232** **(91)**	**21** **(21)**	**59** **(100)**	**36** **(100)**	**47** **(100)**	**97** **(99)**	**253** **(94)**	**196** **(92)**	**296** **(94)**	**239** **(93)**	**24** **(92)**	**62** **(55)**	**27** **(93)**	**36** **(63)**	**531** **(82)**
Learning and applying knowledge	14 (6)	1 (5)	5 (8)	0	0	1 (1)	19 (8)	19 (10)	20 (7)	20 (8)	1 (4)	3 (5)	0	6 (17)	115 (22)
General tasks and demands	19 (8)	1 (5)	0	0	0	1 (1)	11 (4)	11 (6)	17 (6)	17 (7)	0	1 (2)	0	7 (19)	15 (3)
Communication	32 (14)	0	2 (3)	1 (3)	2 (4)	10 (10)	10 (4)	10 (5)	28 (9)	28 (12)	2 (8)	5 (8)	4 (15)	4 (11)	114 (21)
Mobility	6 (3)	8 (38)	3 (5)	4 (11)	1 (2)	15 (15)	107 (42)	51 (26)	108 (36)	52 (22)	13 (54)	4 (6)	6 (22)	5 (14)	87 (16)
Self-care	50 (22)	1 (5)	0	5 (14)	7 (14)	20 (21)	67 (26)	66 (34)	78 (26)	77 (32)	2 (8)	6 (10)	5 (19)	6 (17)	77 (15)
Domestic life	35 (15)	4 (19)	4 (7)	6 (17)	5 (10)	12 (12)	20 (8)	20 (10)	22 (7)	22 (9)	0	8 (13)	2 (4)	1 (3)	29 (5)
Interpersonal interactions and relationships	25 (11)	1 (5)	0	0	0	10 (10)	10 (4)	10 (5)	12 (4)	12 (5)	0	9 (14)	4 (15)	3 (8)	57 (11)
Major life areas	30 (13)	2 (9)	6 (10)	8 (22)	9 (19)	12 (12)	6 (2)	6 (3)	8 (3)	8 (3)	1 (4)	8 (13)	2 (7)	4 (11)	27 (5)
Community, social and civic life	28 (12)	3 (14)	39 (66)	12 (33)	24 (51)	16 (16)	3 (1)	3 (2)	3 (1)	3 (1)	5 (21)	18 (29)	4 (15)	0	10 (2)
**Environmental factors** **(%)**	**2** **(1)**	**0**	**0**	**0**	**0**	**0**	**0**	**0**	**0**	**0**	**0**	**47** **(42)**	**2** **(7)**	**15** **(26)**	**0**
Products and technology	0	0	0	0	0	0	0	0	0	0	0	10 (21)	0	7 (47)	0
Natural environment and human-made changes to the environment	0	0	0	0	0	0	0	0	0	0	0	17 (36)	0	0	0
Support and relationships	1 (50)	0	0	0	0	0	0	0	0	0	0	7 (15)	0	5 (33)	0
Attitudes	0	0	0	0	0	0	0	0	0	0	0	5 (11)	2 (100)	2 (13)	0
Services, systems and policies	1 (50)	0	0	0	0	0	0	0	0	0	0	8 (17)	0	1 (7)	0

^1^ ABAS-3, Adaptive Behavior Assessment System, 3rd edition; AusTOMs-OT, Australian Therapy Outcome Measures for Occupational Therapy, 3rd edition; CAPE-PAC, Children’s Assessment of Paticipation and Enjoyment and Preferences for Activities of Children; COPM, Canadian Occupational Performance Measure; CPQ, Children’s Participation Questionnaire; LIFE-H, Assessment of Life Habits; PEDI-CAT, Pediatric Evaluation of Disability Inventory—Computer Adaptive Test; PEDI-CAT (MD), PEDI-CAT with mobility device; PEDI-CAT ASD, PEDI-CAT module for autism spectrum disorder; PEDI-CAT ASD (MD), PEDI-CAT ASD with mobility device; PEGS, Perceived Efficacy Goal Setting System; ROPP, Rating of Perceived Participation; SCOPE, Short Child Occupational Profile; Vineland-3, Vineland Adaptive Behaviour Scales, 3rd edition.

**Table 6:** Percentage of total codes linking to the International Classification of Functioning, Disability and Health Core Sets [20] for Autism covered by measures of functioning.

	Measures of Functioning ^1^														
	ABAS-3	AusTOMs-OT	CAPE-PAC	COPM	CPQ	LIFE-H	PEDI-CAT	PEDI-CAT (MD)	PEDI-CAT ASD	PEDI-CAT ASD (MD)	PEGS	PEM-CY	ROPP	SCOPE	Vineland-3
Codes (Total)	256	99	59	36	47	98	270	213	315	258	26	112	29	57	648
**Codes linking to Comprehensive ICF ^2^ Core Set for Autism** ***n* (%)**	**242 (95)**	**48** **(49)**	**52** **(88)**	**27** **(75)**	**39** **(83)**	**77** **(79)**	**150** **(56)**	**146** **(69)**	**191** **(59)**	**186** **(72)**	**14** **(54)**	**61** **(55)**	**20** **(69)**	**35** **(61)**	**520** **(80)**
Body functions	21 (8)	42 (88)	0	0	0	1 (1)	13 (9)	13 (9)	15 (8)	15 (8)	2 (14)	0	0	5 (14)	105 (20)
Activities & Participation	220 (91)	6 (12)	52 (100)	27 (100)	39 (100)	76 (99)	137 (91)	133 (91)	176 (92)	171 (92)	12 (86)	42 (69)	18 (90)	18 (51)	415 (80)
Environmental Factors	1 (1)	0	0	0	0	0	0	0	0	0	0	19 (31)	2 (10)	12 (34)	0
**Codes linking to Brief ICF Core Set for Autism (6–16 years)** ***n* (%)**	**156** **(61)**	**35** **(35)**	**43** **(73)**	**18** **(50)**	**30** **(64)**	**49** **(50)**	**104** **(39)**	**102** **(48)**	**132** **(42)**	**129** **(50)**	**10** **(39)**	**43** **(38)**	**11** **(38)**	**34** **(60)**	**418** **(65)**
Body functions	21 (13)	30 (86)	0	0	0	1 (2)	13 (12)	13 (13)	14 (11)	14 (11)	2 (20)	0	0	5 (15)	102 (24)
Activities & Participation	134 (86)	5 (14)	43 (100)	18 (100)	30 (100)	48 (98)	91 (88)	89 (87)	118 (89)	115 (89)	8 (80)	27 (63)	9 (82)	17 (50)	316 (76)
Environmental Factors	1 (1)	0	0	0	0	0	0	0	0	0	0	16 (37)	2 (18)	12 (35)	0

^1^ ABAS-3, Adaptive Behavior Assessment System, 3rd edition; AusTOMs-OT, Australian Therapy Outcome Measures for Occupational Therapy, 3rd edition; CAPE-PAC, Children’s Assessment of Paticipation and Enjoyment and Preferences for Activities of Children; COPM, Canadian Occupational Performance Measure; CPQ, Children’s Participation Questionnaire; LIFE-H, Assessment of Life Habits; PEDI-CAT, Pediatric Evaluation of Disability Inventory—Computer Adaptive Test; PEDI-CAT (MD), PEDI-CAT with mobility device; PEDI-CAT ASD, PEDI-CAT module for autism spectrum disorder; PEDI-CAT ASD (MD), PEDI-CAT ASD with mobility device; PEGS, Perceived Efficacy Goal Setting System; ROPP, Rating of Perceived Participation; SCOPE, Short Child Occupational Profile; Vineland-3, Vineland Adaptive Behaviour Scales, 3rd edition; ^2^ International Classification of Functioning, Disability and Health.

**Table 7:** Percentage of unique codes linking to the International Classification of Functioning, Disability and Health Core Sets [20] for Autism covered by measures of functioning.

	Measures of Functioning ^1^														
	ABAS-3	AusTOMs-OT	CAPE-PAC	COPM	CPQ	LIFE-H	PEDI-CAT	PEDI-CAT (MD)	PEDI-CAT ASD	PEDI-CAT ASD (MD)	PEGS	PEM-CY	ROPP	SCOPE	Vineland-3
**Unique codes linking to Comprehensive ICF ^2^ Core Set for Autism** ***n* (%) (110)**	**48** **(44)**	**10** **(9)**	**11** **(10)**	**13** **(12)**	**12** **(11)**	**30** **(27)**	**44** **(40)**	**43** **(38)**	**46** **(42)**	**45** **(41)**	**14** **(13)**	**23** **(21)**	**18** **(16)**	**28** **(25)**	**67** **(61)**
Body functions (20)	8 (40)	4 (20)	0	0	0	1 (5)	2 (10)	2 (10)	2 (10)	2 (10)	2 (10)	0	0	4 (20)	17 (85)
Activities & Participation (59)	39 (66)	6 (10)	11 (19)	13 (22)	12 (20)	29 (49)	42 (71)	40 (68)	44 (75)	43 (73)	6 (10)	14 (24)	16 (27)	14 (24)	50 (85)
Environmental Factors (31)	1 (3)	0	0	0	0	0	0	0	0	0	0	9 (29)	2 (6)	10 (32)	0
**Unique codes linking to Brief ICF Core Set for Autism (6–16 years)** ***n* (%) (81)**	**31 (38)**	**7** **(9)**	**4** **(5)**	**6** **(7)**	**5** **(6)**	**16** **(20)**	**28** **(35)**	**28** **(35)**	**29** **(36)**	**29** **(45)**	**10** **(12)**	**15** **(19)**	**8** **(10)**	**27** **(33)**	**47** **(58)**
Body functions (18)	8 (44)	3 (17)	0	0	0	1 (6)	2 (11)	2 (11)	2 (11)	2 (11)	2 (11)	0	0	4 (22)	16 (89)
Activities & Participation (36)	22 (61)	4 (11)	4 (11)	6 (17)	5 (14)	15 (42)	26 (72)	26 (72)	27 (75)	27 (75)	3 (8)	7 (19)	7 (19)	13 (36)	31 (86)
Environmental Factors (27)	1 (4)	0	0	0	0	0	0	0	0	0	0	8 (30)	1 (4)	10 (37)	0

^1^ ABAS-3, Adaptive Behavior Assessment System, 3rd edition; AusTOMs-OT, Australian Therapy Outcome Measures for Occupational Therapy, 3rd edition; CAPE-PAC, Children’s Assessment of Paticipation and Enjoyment and Preferences for Activities of Children; COPM, Canadian Occupational Performance Measure; CPQ, Children’s Participation Questionnaire; LIFE-H, Assessment of Life Habits; PEDI-CAT, Pediatric Evaluation of Disability Inventory—Computer Adaptive Test; PEDI-CAT (MD), PEDI-CAT with mobility device; PEDI-CAT ASD, PEDI-CAT module for autism spectrum disorder; PEDI-CAT ASD (MD), PEDI-CAT ASD with mobility device; PEGS, Perceived Efficacy Goal Setting System; ROPP, Rating of Perceived Participation; SCOPE, Short Child Occupational Profile; Vineland-3, Vineland Adaptive Behaviour Scales, 3rd edition; ^2^ International Classification of Functioning, Disability and Health.

## Data Availability

The data presented in this study are not publicly available.

## Appendix A

**Table A1:** Search Terms Used to Identify Relevant Articles.

Search terms relating to psychometric properties
**(psychometric * OR validity OR “predictive validity” OR “internal validity” OR “face validity” OR “external validity” OR “discriminant validity” OR “criterion related validity” OR “concurrent validity” OR “construct validity” OR “content validity” OR “validation study” OR reliability OR “reliability and validity” OR “test–retest reliability” OR “intrarater reliability” OR “interrater reliability” OR psychometric* OR reliabil* OR valid* OR sensitivity OR specificit* OR bias OR reproducib* OR feasib* OR “clinical utility” OR usability OR appropriate* OR accessib* OR practiab* OR acceptab*)**
AND
Assessment specific terms
Adaptive Behavior Assessment System
**(“adaptive behavior assessment system” or “ABAS”)**
Australian Therapy Outcome Measures for Occupational Therapy
**(“Australian Therapy Outcome Measures for Occupational Therapy” or “AusTOMs-OT” or “AusTOMS OT”)**
Canadian Occupational Performance Measure
**(“Canadian Occupational Performance Measure” OR COPM)**
Children’s Assessment of Participation and Enjoyment and Preferences for Activities of Children (CAPE-PAC)
**(“Children’s assessment of participation and enjoyment” or CAPE or “Enjoyment and preferences for activities of children” or “CAPE-PAC”)**
Children’s Participation Questionnaire
**(“Children’s participation questionnaire” or CPQ)**
Life Habits Assessment
**(“Life habits assessment” or “LIFE H” or “LIFE-H”)**
PEM-CY
**(pem-cy OR “participation and environment measure” OR “participation and environment measure children and youth”)**
PEDI-CAT/PEDI-CAT (ASD)
**(pedicat OR “pedicat asd” OR pedi-cat OR “pediatric evaluation of disability inventory computer adaptive test”)**
Perceived Efficacy and Goal Setting
**(“Perceived efficacy and goal setting” or PEGS)**
Rating of Perceived Participation
**(“Rating of perceived participation” or ROPP)**
Short Child Occupational Profile
**(“Short child occupational profile” or SCOPE)**
Vineland-3
**(vineland OR vineland-3 OR “vineland three” OR “vineland III” OR “vineland 3” OR vineland-III OR “vineland third edition”**

* Indicates a truncation wildcard, used to search for variations of the core word
